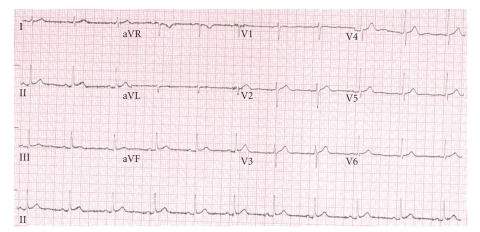# Hypercalcaemia Mimicking STEMI on Electrocardiography

**DOI:** 10.1155/2010/563572

**Published:** 2010-12-16

**Authors:** Joseph Donovan, Mark Jackson

**Affiliations:** Royal Sussex County Hospital, Brighton BN2 5BE, UK

## Abstract

Acute coronary syndrome is a common cause of presentation to hospital. ST segment elevation on an electrocardiogram (ECG) is likely to be cardiac in origin, but in low-risk patients other causes must be ruled out. We describe a case of a man with hypercalcaemia, no evidence of cardiac disease, and ECG changes mimicking acute myocardial infarction. These ECG changes resolved after treatment of the hypercalcaemia.

## 1. Introduction

Acute coronary syndrome is a common cause of presentation to hospital emergency departments. In patients who present with chest pain, ST segment elevation is likely to be cardiac in origin and prompt recognition and treatment improves outcomes. However, unnecessary treatment with thrombolytic therapy or anticoagulation can be harmful, and in patients who are at low risk of cardiac disease other causes must be ruled out. Hypercalcaemia may be caused by a variety of illnesses and can present acutely with a range of symptoms. Hypercalcaemia is usually reversible with intravenous fluid and bisphosphonate whilst the cause is simultaneously investigated.

## 2. Case Report

A 39-year-old man presented to the acute medical team with generalised weakness, vomiting, constipation, and abdominal pain. He had no chest pain or shortness of breath. The patient had no prior cardiac history. He had a 40-pack year smoking history, but he was not known to be hypertensive and there were no other risk factors for coronary artery disease. Blood pressure on admission was 105/55. On clinical examination the patient appeared dehydrated. He had a Glasgow Coma Score (GCS) of 13 and was unable to give a clear history at the time of presentation. There was mild epigastric tenderness but without rigidity or guarding. Heart sounds were normal, and there was no evidence of cardiac failure. There were no other significant findings or untoward features.

Blood testing revealed acute renal failure; urea was 21.7 mmol/L and creatinine was 338 *μ*mol/L. His plasma adjusted calcium was 5.75 mmol/L and his albumin was 38 g/L, and parathyroid hormone was suppressed at 9 ng/L (normal range: 15–65 ng/L). Chest radiography revealed no features of malignancy or left ventricular failure, and a myeloma screen was negative. Thyroid function tests were also normal.

**Figure 1:**
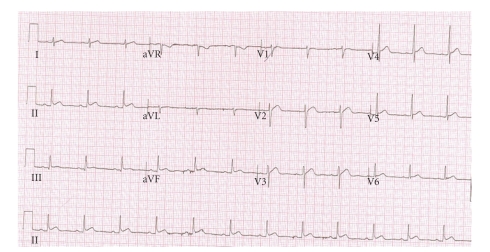


The ECG at presentation (Figure 1) revealed abnormal ST morphology in leads II, aVF, and V2-V3. These changes were minimal, and thrombolysis was not indicated.

The patient underwent initial resuscitation with intravenous fluids, and subsequently intravenous pamidronate was administered to correct the hypercalcaemia. His condition improved rapidly, and he was subsequently able to provide a detailed history. This revealed that he had been taking an over-the-counter calcium carbonate supplement: Tums. He had been ingesting extremely large quantities, up to 112 g calcium carbonate daily, for six months. This medication had initially been taken for indigestion. The patient was unaware of the detrimental effects these supplements could have on his health. Repeating a review of systems did not elicit any other significant symptoms, and there were no features suggestive of malignancy. Blood pressure recording on the ward at discharge was 128/70. An ECG following reversal of the hypercalcaemia (Figure 2) showed resolution of the ST segment elevation.

## 3. Discussion

Severe hypercalcaemia provoking ECG changes mimicking acute myocardial infarction is infrequently reported. It is important for physicians to recognise severe hypercalcaemia as a differential diagnosis for ST segment elevation on the ECG. Wesson et al. described this association, in a patient with a past medical history of ischaemic heart disease, coronary angioplasty, hypertension, and left ventricular failure [1]. Subsequent comments about this case suggested that the ST changes might have been due to a left ventricular aneurysm [2, 3]. 

Shawn et al. described a case of a patient with ST segment elevation induced by hypercalcaemia [4]. Resolution of ST segment elevation occurred on correction of the hypercalcaemia. A transthoracic echocardiogram demonstrated that there was underlying moderate left ventricular hypertrophy, and the patient had an ejection fraction of 50%.

Our patient had no history of cardiac disease or hypertension. He was a smoker, but there were no other risks for cardiac disease. His acute ST changes on ECG resolved on reversal of his hypercalcaemia.

Our case forms a case series with previous cases [1, 5–8], demonstrating a clear link between hypercalcaemia and ECG changes mimicking acute myocardial infarction. Our case also underlines the importance of awareness of overuse of over-the-counter supplements.

##  Conflict of Interests

The authors declare no conflict of interests. Patient Consent was obtained.

## Figures and Tables

**Figure 2:**